# Circling in on Convective Self‐Aggregation

**DOI:** 10.1029/2021JD035331

**Published:** 2021-10-11

**Authors:** Silas Boye Nissen, Jan O. Haerter

**Affiliations:** ^1^ Niels Bohr Institute University of Copenhagen Copenhagen Denmark; ^2^ Physics and Earth Sciences Jacobs University Bremen Bremen Germany; ^3^ Leibniz Center for Tropical Marine Research Complexity and Climate Bremen Germany

**Keywords:** cloud‐free region, cold pools, convection, convective self‐aggregation, gust fronts, precipitation

## Abstract

In radiative‐convective equilibrium simulations, convective self‐aggregation (CSA) is the spontaneous organization into segregated cloudy and cloud‐free regions. Evidence exists for how CSA is stabilized, but how it arises favorably on large domains is not settled. Using large‐eddy simulations, we link the spatial organization emerging from the interaction of cold pools (CPs) to CSA. We systematically weaken simulated rain evaporation to reduce maximal CP radii, $R_{\text{max}}$, and find reducing $R_{\text{max}}$ causes CSA to occur earlier. We further identify a typical rain cell generation time and a minimum radius, $R_{\text{min}}$, around a given rain cell, within which the formation of subsequent rain cells is suppressed. Incorporating $R_{\text{min}}$ and $R_{\text{max}}$, we propose a toy model that captures how CSA arises earlier on large domains: when two CPs of radii $r_{i},r_{j}\in [R_{\text{min}},R_{\text{max}}]$ collide, they form a new convective event. These findings imply that interactions between CPs may explain the initial stages of CSA.

## Key Points

- Smaller cold pool radii in large‐eddy simulations diminish the time to reach convective self‐aggregation
- We report cold pools' generation time and suppression radius by evaluating the distance between rain events connected in time
- A mathematical model captures the effect of domain size, suppression radius, and maximum cold pool radius in convective self‐aggregation

## 1 Introduction

When evaporation of rain from convective clouds is strong, so is the associated sub‐cloud cooling and density increase (Engerer et al., 2008; Simpson, 1980), forcing the resulting cold pools (CPs) to spread more quickly and cover larger areas (Romps & Jeevanjee, 2016; Torri et al., 2015; Zuidema et al., 2017). Such pronounced CP activity has repeatedly been suggested to hamper convective self‐aggregation (CSA) in radiative‐convective equilibrium (RCE) numerical experiments (Holloway & Woolnough, 2016; Hohenegger & Stevens, 2016; Jeevanjee & Romps, 2013; Muller & Bony, 2015; Yanase et al., 2020). In these simulations, the atmosphere gradually organizes from an initially homogeneous population of convective updrafts into a segregated pattern with strongly convecting regions and dry, precipitation‐free regions (Bretherton et al., 2005; Held et al., 1993; Hohenegger & Stevens, 2016; Tompkins & Craig, 1998; Wing et al., 2017).

Generically, CSA is characterized by the appearance of long‐lived dry and warm patches within which cloud and rain are suppressed (Holloway et al., 2017). Further drying increasingly occurs through enhanced radiative cooling in already dry regions and the resulting subsidence. Later, the dry regions expand and merge, eventually leaving only one contiguous moist area with intense low‐level convergence feeding convection. Surface latent and sensible heat fluxes, which increase under stronger surface wind speed, may further increase low‐level moisture convergence.

Physically, CPs spread as density currents along the surface, carry kinetic energy and buoyancy, and modify the thermodynamic structure near the CP edges (de Szoeke et al., 2017; Langhans & Romps, 2015; Tompkins, 2001). Thereby, CPs spatially organize the convectively unstable atmosphere, establishing connections between the loci where new convective cells emerge and loci at which the previous cells dissipated. In particular, new cells were suggested to be triggered by the CP gust front alone or by collisions between gust fronts (Cafaro & Rooney, 2018; de Szoeke et al., 2017; Fuglestvedt & Haerter, 2020; Glassmeier & Feingold, 2017). Inspired by the notion of CP interactions, CP representations have been incorporated into large‐scale models (Grandpeix & Lafore, 2010), and conceptual work has formulated CPs as cellular automata (Böing, 2016; Haerter et al., 2020; Windmiller, 2017). Recent work addressed the diurnal cycle of convection, where CPs effectively increased the typical length scale in the cloud field (Haerter et al., 2019). In such an out‐of‐equilibrium context, mechanical lifting upon collisions of three CPs was found a dominant process, as moist boundary layer air, enclosed by gust fronts laterally, was forced to escape vertically. Triggering of new convection in such situations occurs rapidly, usually within one hour after the collision. As was shown, the three‐CP collision model inevitably leads to decreased CP population over time. In RCE, slow thermodynamics processes at gust front collisions are more typical (Tompkins, 2001; Fuglestvedt & Haerter, 2020). Before the onset of CSA, length scales and CP numbers are approximately conserved over time. A simple model, discussed below, capable of capturing such conserved length scales requires collisions between two rather than three CPs.

Studies on CSA often argue that sufficiently large domain sizes ($\geq 500\times 500\;{km}^{2}$) and coarse horizontal resolutions (≥2 km) are required for CSA (Bretherton et al., 2005; Muller & Bony, 2015; Yanase et al., 2020). To examine this claim more closely, for deliberately small domain sizes ($96\times 96\;{km}^{2}$) and fine horizontal resolution (200 m), we show that CSA sets in earlier when CPs are weakened through reductions in rain evaporation, that is, when the CP maximal radius, which we term $R_{\text{max}}$, is reduced. We track the CP gust fronts to motivate that loci of gust front collisions are preferable for subsequent convective rain cells. Dependent on rain evaporation, we further detect a minimal distance $R_{\text{min}}$, effectively an updraft suppression radius, within which subsequent rain cells are unlikely to form, as well as a typical rain cell generation time. Using these findings, we build, simulate, and analyze a simple mathematical model, which helps understand CSA formation. We explore this model's phase diagram and find that the transition into convecting and nonconvecting subregions occurs later for large $R_{\text{max}}$, small $R_{\text{min}}$, or small domain sizes L.

## 2 Materials and Methods

### 2.1 Large‐Eddy Simulations

**Figure 1:**
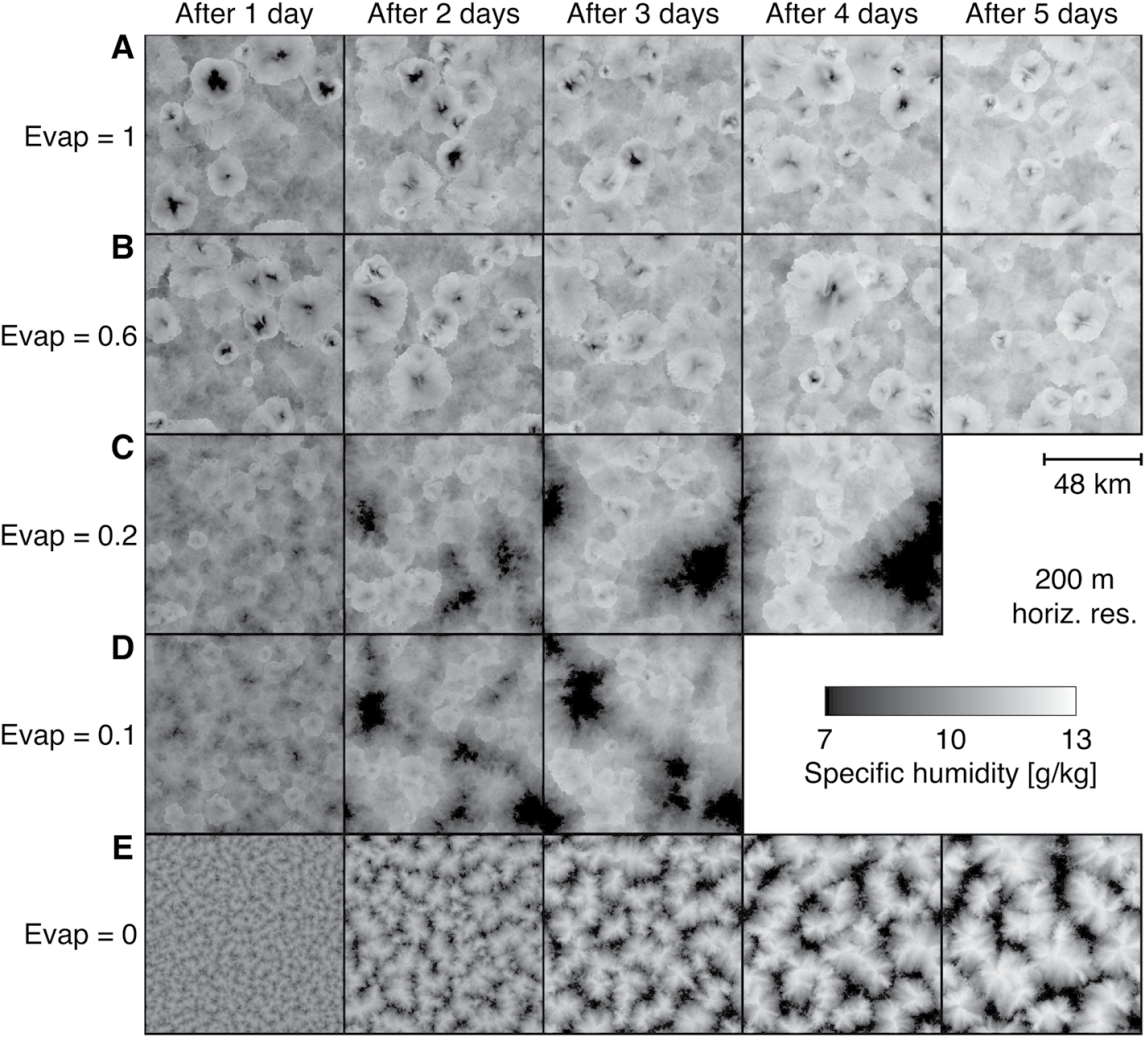
The onset of convective self‐aggregation. Near‐surface specific humidity $q_{v}$(50 m) the first 10 min of each simulation day in radiative‐convective equilibrium simulations with various degrees of rain evaporation. (a) Realistic rain evaporation (control simulation). (b) 60%, (c) 20%, (d) 10%, and (e) 0% rain evaporation relative to (a). Note the pronounced moisture reduction in (a and b) and the weakened moisture reduction in (c and d) within cold pool centers. Further note the evolving moisture segregation, typical of convective self‐aggregation (c and d) and moisture coarsening progression (e).

We conducted a suite of simulations on a (96 km)^2^ domain using the University of California, Los Angeles (UCLA) Large Eddy Simulator. The horizontal model grid is regular, and horizontally periodic boundary conditions are applied in both lateral dimensions. Vertical model resolution varies from 100 m below 1 km, stretching to 200 m near 6 km, and finally 400 m in the upper layers with 75 vertical levels in total. The Coriolis force and the mean wind were set to zero with weak, spatially uncorrelated random initial temperature perturbations, sampled uniformly within [−0.2,0.2] K for each grid box, added as noise to break complete spatial symmetry. At each output time step of 10 min, instantaneous surface precipitation intensity, specific humidity, temperature, liquid water mixing ratio, and 3D velocities are output at various model levels. We used sub‐grid scale turbulence parametrized after Smagorinsky (1963), delta four‐stream radiation (Pincus & Stevens, 2009), and a two‐moment cloud microphysics scheme (Stevens et al., 2005). Rain evaporation is accounted for by Seifert and Beheng (2006). The five simulations have identical setups, except that the ventilation coefficient for hydrometeors is varied by fractions {1.0,0.6,0.2,0.1,0} of its default over all vertical layers, thus influencing the rate of re‐evaporation. In the following, these simulations are correspondingly labeled as “Evap = 1,” “Evap = 0.6,” etc. All simulations are run for five days, except Evap = 0.2 that runs for four days, and Evap = 0.1 that runs for three days (see Figure 1). In both Evap = 0.2 and Evap = 0.1, the onset of CSA could already be distinguished after such shorter periods. Surface temperatures are set constant to 300 K, and insolation is fixed using a constant equatorial zenith angle of 50° to a constant 655 W $m^{-2}$ (Bretherton et al., 2005). Surface latent and sensible heat fluxes are computed interactively and depend on the vertical temperature and humidity gradients and horizontal wind speed (bulk formula), approximated using the Monin‐Obukhov similarity theory. Surface latent heat fluxes are set to 70 percent of those for a water surface. Temperature and humidity are initialized using horizontal‐mean vertical profiles of temperature and humidity obtained from a prior approximately three‐day spin‐up using 400 m horizontal resolution (see Figure S1). To explore resolution effects, we supplemented these simulations using the settings of Evap = 1 repeated using horizontal resolutions of 1 km, 2 km, and 4 km, each maintaining the number of 480×480 horizontal grid boxes.

### 2.2 Tracking of Cold Pools

**Figure 2:**
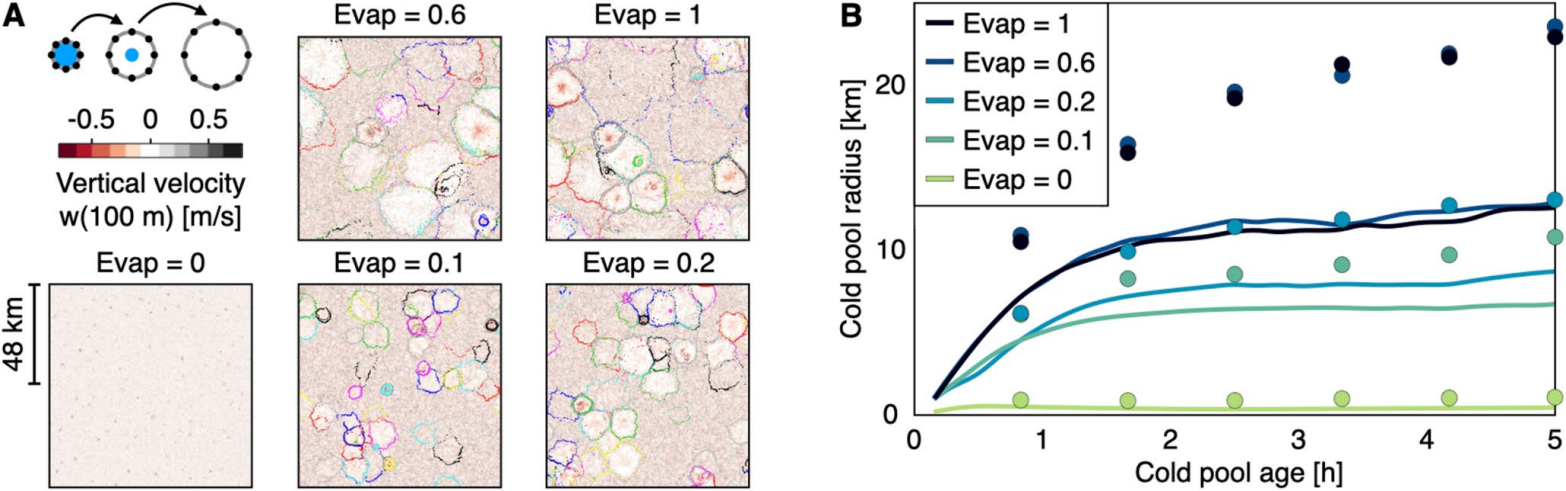
Maximum radius, ${\text{R}}_{\text{max}}$. (a) Tracking all cold pool (CP) gust fronts present during the first 18 h of precipitation. Top‐left cartoon: we track a CP gust front (gray rim) by placing tracers (black points) around the rain event (blue spot) and let the tracers move radially away from the rain event with the horizontal wind (Details*:* Methods). Each panel shows the near‐surface vertical velocity field 18 h after precipitation onset and gust front tracers marked by colors indicating different CPs. (b) Composite (average) CP radii as the CPs evolve after their emergence (lines) and the 90th radius percentile (dots). Note that CPs initially grow quickly but monotonically slow and that the maximal CP radii increase with rain evaporation rate.

**Figure 3:**
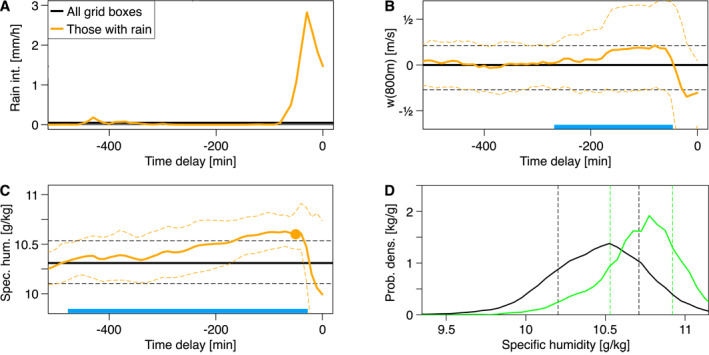
Identifying cold pool collisions. The time t=0 is defined as 1,670 min after model initialization, a time point sufficiently close to the onset of precipitation in Evap = 1 (≈600 min) but sufficiently long after the onset so that the histories of rain intensity, vertical velocity, and specific humidity can be tracked. (a) History of rain intensity conditionally averaged over all grid boxes with rainfall at t=0 (orange) and domain mean rainfall (black) for the simulation Evap = 1. (b) Analogous to A, but for vertical velocity near the cloud base (w[800 m]). The domain average is zero throughout. Thin lines mark corresponding 20th and 80th percentiles. The blue bar highlights the time during which updrafts exceed the domain average. Note the pronounced peak, corresponding to convective updrafts, as is expected before rain onset, and the dip near t=0, corresponding to CP‐associated downdrafts. (c) Analogous, but for near‐surface specific humidity, $q_{v}$(z = 50 m). The blue bar highlights the time during which specific humidity exceeds the domain average. Note the relatively long build‐up of humidity before rainfall onset. (d) Histograms of $q_{v}$(50 m) at t=0 for all data (black curve) and gust front positions only (green curve).

To track CP gust fronts, we follow the tracer particle methodology described in the literature (Haerter et al., 2019; Henneberg et al., 2020) using a threshold of $I_{0}\equiv$ 0.5 mm $h^{-1}$ for the rain intensity within the initial surface precipitation patch. As this tracking method is implemented to run “offline,” it uses only the recorded discrete 10 min output time steps of precipitation intensity I and lowest‐level horizontal wind velocity (u[50 m], v[50 m]). To compare the temporal evolution of CP radii transparently between the different simulations (Figure 2), we consider that the time of rainfall onset slightly differs between the simulations (compare curves in Figure S2b). We define the time of rainfall onset as the first time point where one or more pixels have $I>I_{0}$. We then track all CP gust fronts present during the following 18 h. Each CPs is followed for five hours, and the start time of all tracked CPs is aligned to produce composite statistics. The time interval of 18 h was found sufficient to yield significant statistics on the spreading of each CP but short enough so that not many CP collisions were encountered. Conversely, to study collision effects (Figure 3), we used a late‐stage (∼4 days after initialization) of the control simulation (Evap = 1). For Evap = 1, CP radii are large, and CPs are thus space‐filling. Therefore, any new CP inevitably collides with recent CPs in its surroundings.

### 2.3 Mathematical Model

**Figure 4:**
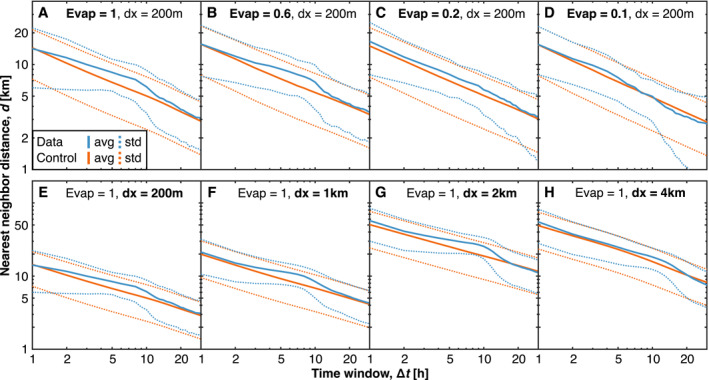
Generation time and an effective minimal radius, ${\text{R}}_{\text{min}}$. (a–d) The average distance (d) between rain events occurring within the first 12 h past precipitation onset and their nearest neighbor rain event (blue) occurring within a time window (Δt) for varying evaporation rates (Evap). We contrast that to a control where the same number of rain events are located uniformly (red). The dotted lines mark the standard deviations. More rain events are included for larger time windows, causing the distances to be smaller. In (a), note the lack of events within 6 km for up to 8 h. (e–h) Analog, but for varying horizontal resolutions (dx). Note the inhibitory distance increases for coarser horizontal resolutions without changing the time scale at which it occurs.

The mathematical model can be described in two sentences: (a) The initial conditions: $N_{1}$ randomly located points on a 2D domain of size L×L with double‐periodic boundary conditions expand into circles (representing cold pools) with equal and constant radial speed, $v_{0}$. (b) The dynamics: When two circles meet, both having their radii lie between $R_{\text{min}}$ and $R_{\text{max}}$ (justified in Figures 4 and 2, respectively), the two circles instantly, at their first intersection point, initiate a new point that expands with the same radial speed, $v_{0}$. In other words, this model follows the principles in Haerter et al. (2019) with the major difference that two, instead of three, circles can initiate the growth of a new circle.

**Figure 5:**
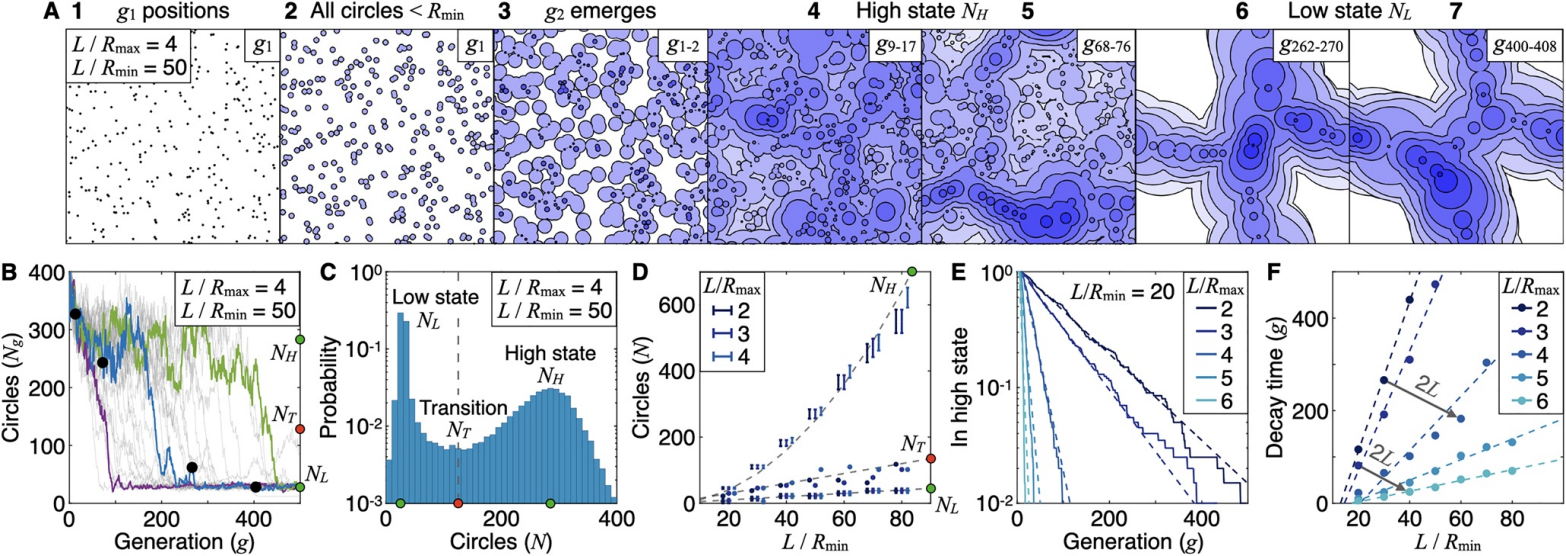
Circle model. (a) Seven snapshots running forward in time from one model run with $L/R_{\text{max}}$ = 4 and $L/R_{\text{min}}$ = 50. Snapshots 1–2 show the initial $g_{1}$ positions. Snapshot 3 shows the emergence of $g_{2}$ circles (cold pools). Snapshots 4–5 and 6–7 show representative pictures of the high state ($N_{H}$) and the low state ($N_{L}$), respectively (Details*:* Methods). Within each snapshot, circles of the same color belong to the same generation (g), and white (dark blue) areas correspond to the most historic (recent) generations. (b) The number of circles ($N_{g}$) per generation (g) as 30 simulations with varying initial $g_{1}$ positions evolve. Three runs are highlighted. The blue curve represents the simulation in (a), and the black dots indicate the time points of the last four snapshots in (a). Note the existence of two qualitatively distinct states of high and low $N_{g}$ marked with green dots as $N_{H}$ and $N_{L}$, respectively. The red dot, $N_{T}$, marks the transition point. (c) The distribution of circles in all generations pooled together for 200 runs. In (a–c), $L/R_{\text{max}}$ = 4 and $L/R_{\text{min}}$ = 50. (d) The number of circles in the low $N_{L}$ and high $N_{H}$ states and at the transition point $N_{T}$ for varying $L/R_{\text{max}}$ and $L/R_{\text{min}}$. (e) The fraction of runs in the high state as generation number for $L/R_{\text{min}}$ = 20. Note the logarithmic vertical axes. (f) The characteristic decay time as a function of $L/R_{\text{max}}$ and $L/R_{\text{min}}$. Note that higher L, higher $R_{\text{min}}$, or lower $R_{\text{max}}$ result in faster decay.

The outcome of this model is nontrivial. Since all circles expand with equal and constant speed, $v_{0}$, the dynamics allow us to categorize circles into independent generations that mathematically cannot interact with each other. To realize this, let us go through one example: In Figure 5a snapshot 1, the initially $N_{1}$ seeded points constitute generation one, denoted as $g_{1}$. At slightly later snapshot 2, these points have expanded into equally sized circles that are all smaller than $R_{\text{min}}$ and therefore do not trigger the growth of a new circle when they collide. In snapshot 3, all $g_{1}$ circles have grown beyond $R_{\text{min}}$, and some have collided and initiated the start of $g_{2}$ circles. Since both generations continue to expand with $v_{0}$, circle areas corresponding to generation 2 will always lie within areas corresponding to generation 1. Therefore, in general, a $g_{i+1}$ circle cannot interact with a $g_{i}$ circle.

Mathematically, we define the center of the circlei as $\left[x_{i},y_{i}\right]$ and its increasing radius as $r_{i}$. Thereby, two circles, i and j, are described by the following set of quadratic equations

(1) $${\left(x-x_{i}\right)}^{2}+{\left(y-y_{i}\right)}^{2}=r_{i}^{2}$$

and

(2) $${\left(x-x_{j}\right)}^{2}+{\left(y-y_{j}\right)}^{2}=r_{j}^{2}.$$

The collision point between the circles i and j is described by adding the distance dr to both of their radii, giving

(3) $${\left(x-x_{i}\right)}^{2}+{\left(y-y_{i}\right)}^{2}={\left(r_{i}+dr\right)}^{2}$$

and

(4) $${\left(x-x_{j}\right)}^{2}+{\left(y-y_{j}\right)}^{2}={\left(r_{j}+dr\right)}^{2}.$$

In the model, only collisions that fall onto the straight line between the two circle centers are allowed, assuming that this is the collision point with the highest momentum transfer, thus yielding

(5) $$y=\frac{x-x_{i}}{x_{j}-x_{i}}\left(y_{j}-y_{i}\right)+y_{i}.$$

Equations (2), (3), (4) have three unknowns (x,y,dr) and two solvable solutions that analytically describe the location (x,y) of the collision point and when it occurs (dr). One of these two solutions can be ruled out because it is either negative or nonreal and, therefore, irrelevant to the model.

To implement this model computationally, we consider circles belonging to one generation at a time. For each generation, we find the pairwise distance between all circle centers. For circles whose pairwise distance is larger than $2R_{\text{min}}$ and smaller than $2R_{\text{max}}$, we calculate their mutual collision point by solving Equations (2), (3), (4) above. We sort all collision points by dr and update the system by inserting circles at collision points that fulfill $R_{\text{min}}<r_{i},r_{j}<R_{\text{max}}$, and are not inside a circle belonging to the current generation. When proceeding to the next generation, we return to the time point when the current generation's first circle was seeded. We note that a circle may collide with multiple circles until it reaches $R_{\text{max}}$, and when that occurs, the circle has no further effect. We run the model until circle generation number 500, which corresponds to roughly 5000 h (>200 days) given our measure of a CP generation time (∼10 h) in Figure 4 and a similar finding in Fuglestvedt and Haerter (2020).

In Figure 5, all simulations start with $N_{1}=L^{2}/\left(10R_{\text{min}}^{2}\right)$ points. In the Supporting Information S1, we show that the model results are independent of the particular choice of $N_{1}$ (Figure S3). Besides, we derive that given $R_{\text{min}}=0$ and $R_{\text{max}}=\infty$, an initial random generation‐one population $N_{1}$ of circles would yield $N_{2}=2N_{1}$ (Supporting Information S1). Subsequent growth of $N_{g}$ versus g would be nearly exponential. Although the lack of synchronous circle expansion beyond the first generation leads to a slight reduction of replication rate, the rate eventually stabilizes near 1.9 (Figure S4). In other words, super‐exponential growth in time would occur for $R_{\text{min}}=0$ and result in singularities, that is, infinitely rapid nonrealistic replication.

## 3 Results

### 3.1 Weakening Cold Pools in RCE Simulations Speeds up the Onset of Self‐Aggregation

A control simulation with realistic rain evaporation (Figure 1a) shows no indication of CSA. We check this by computing the interquartile specific humidity difference (Figure S2a), finding a weak initial increase when the first CPs set in, but we find no further increase over time. While leaving the total number of rain cells and domain‐average rainfall approximately unchanged (Figures S2b and S5a), decreasing the rate of rain evaporation (Figures 1b–1e) yields a monotonic increase in humidity variation (Figure S2a) and overall higher near‐surface temperature (Figure S2c), along with a systematically earlier onset of persistent dry patches, for example, near day 2 for Evap = 0.2 (Figure 1c). This comparison underlines findings from Jeevanjee and Romps (2013) and Muller and Bony (2015), who reported that CPs hamper self‐aggregation. The five experiments highlight that reducing rain evaporation weakens subsidence drying in the center of CPs (compare dark spots in Figures 1a and 1b vs. 1c and 1d) and visibly reduces CP radii. We also note that intermediate values of evaporation appear to allow for a band‐like aggregation state, where rain cells form a quasi‐one‐dimensional chain around one of the horizontal dimensions (Figure 1c on day 4). When rain evaporation is entirely removed (Figure 1e), any organizing effect through CPs is absent: one is left with a coarsening process akin to reaction‐diffusion dynamics (Craig & Mack, 2013; Windmiller & Craig, 2019), small impurities gradually merging into larger structures.

### 3.2 Measuring the Maximum Cold Pool Radius, ${\text{R}}_{\text{max}}$

Using a rain cell (Moseley et al., 2019) and CP (Haerter et al., 2019; Henneberg et al., 2020) tracking method, we seed tracer particles at the boundary of surface rain patches (Figure 2a, top‐left cartoon). We advect these tracers using the radial velocity field, forcing them to gather in pronounced convergence areas caused by the CP gust fronts (Details*:* Methods). Superimposing the resulting pattern of tracers onto the near‐surface vertical velocity field (Figure 2a) confirms that the tracers gather along the edge of each CP (subsident or featureless vertical wind field). By plotting the average time evolution of the CP radii in each simulation (Figure 2b), we find that for Evap = 1, CPs on average expand to 11 km 5 h after initiation with the 90th radius percentile reaching 23 km. This value is comparable to previous simulation results found on various domain sizes (Romps & Jeevanjee, 2016; Tompkins, 2001) and observational findings (Black, 1978; Feng et al., 2015; Zuidema et al., 2012). Reducing evaporation results in systematically smaller CP radii: for Evap = 0.2, CPs on average reach 8 km in radii during the same time with 90th percentile reaching 13 km, and for Evap = 0, CP radii equal the corresponding surface rain cells, as, without CPs, there is no pronounced wind field to advect the tracers.

### 3.3 New Convective Events Are Initiated in the Vicinity of Cold Pool Collisions

What is then the specific role of CPs in maintaining domain‐wide convection? To explore this, first consider locations of rainfall at a particular time step of Evap = 1 (Figure 3a), the associated cloud‐base vertical velocity (Figure 3b), and specific humidity (Figure 3c). Updrafts form shortly before the onset of rainfall. In contrast, specific humidity becomes elevated earlier, in line with RCE simulations, where a considerable moisture build‐up before any subsequent convective event was reported (Fuglestvedt & Haerter, 2020). Second, we determine gust front loci using CP tracer particles, which have been shown to gather at the intersections between CPs (Haerter et al., 2019; Henneberg et al., 2020). The humidity during rain event build‐up (peak highlighted in Figure 3c) is elevated by ∼0.3–0.4 g/kg compared to the domain mean (bold horizontal line in the panel). Using the tracers to collect, as a comparison, the specific humidity at CP gust fronts, it is found that this histogram is similarly shifted to moister values (Figure 3d, compare green vs. black curve). In summary, loci of CP collisions do provide the positive humidity anomalies typical of subsequent convective events.

### 3.4 New Deep Convective Events Are Initiated at a Certain Distance, ${\text{R}}_{\text{min}}$, Away From Earlier Events

To quantify a possible suppression effect caused by a present rain cell's CP on subsequent cells forming within the surroundings, we examine whether rain events are spaced uniformly after the initial rain onset. A nonuniform spacing would imply either suppression (larger distance) or activation (smaller distance), whereas a uniform spacing would speak against a direct spatial influence on subsequent rain cell formation. We thus identify all rain events within the first 12 h after rain onset (Moseley et al., 2013), allowing us to compare non‐aggregating simulations with aggregating simulations (day 1 in Figure 1). We measure each rain cell's distance to its nearest rain event occurring within a time window Δt.

As a control, we use that the probability for n points to all lie outside a circle of radius d is ${\left(1-\pi d^{2}/L^{2}\right)}^{n}$, where L is the domain length and n is the number of rain events during Δt. Differentiating this with respect to −d gives the probability density function $f\left(d\right)=2n\pi d{\left(1-\pi d^{2}/L^{2}\right)}^{n-1}/L^{2}$, from which we compute the expected nearest‐neighbor distance given a uniform distribution of points (orange curves in Figure 4). Comparing this to the simulation data (blue curves in Figure 4), we find an inhibitory effect causing the nearest neighbor distance to be larger than 5 km for up to 8 h. We refer to this distance as $R_{\text{min}}$ ≈ 6 km and explain it by CPs being too negatively buoyant to initialize new convective cells within this distance (Drager & van den Heever, 2017; Fournier & Haerter, 2019). We find that this spatial scale is independent of the rain evaporation rate (Figures 4a–4d), but it increases for coarser horizontal resolutions (Figures 4e–4h). A possible explanation for the latter lies in decreasing rain event number densities (Figure S5b). A caveat in quantifying rain event number densities lies in using a proper definition of rain events at different model resolutions. A common intensity threshold, as we have pragmatically used here, can be debated. Tompkins and Semie (2017) performed a similar nearest neighbor analysis on RCE simulations with a 2 km horizontal resolution. They found that CPs suppress rain events within 20 km of range, supporting our results in Figure 4g. Besides denoting the suppression at small timescales (Δt≲10h) to CPs, they further assigned the activation at larger timescales (Δt≳10h) to CSA, an effect we see most clearly in Figure 4d, which aggregates within three days (Figure 1d).

When increasing the time window of included events beyond Δt=10 h, we find that this suppression effect diminishes; that is, the distribution function approaches a uniform distribution (Figures 4 and S6). On this time scale, the CPs associated with two rain events have time to grow larger than $R_{\text{min}}$, collide, and trigger the formation of a new, closer rain event belonging to the subsequent generation. Therefore, we interpret the time scale ∼10 h as the generation time of one CP. Our data indicate that this time scale slightly decreases for decreasing evaporation rates, likely because those simulations are transitioning to CSA (Figures S6a–S6d). Many rain events must be initiated within a relatively small area in the self‐aggregated state, thus locally driving up the frequency at which new cells are generated. Finally, we find that the same time scale slightly increases for coarser horizontal resolution, likely due to higher average rain intensities per rain event triggering stronger CPs that last for a longer time (Figures S6e–S6h).

### 3.5 A Simple Mathematical Model Captures the Onset of Self‐Aggregation

To understand the role of CP collisions, we introduce a model consisting of growing and colliding circles that represent the gust fronts of CPs (Details*:* Methods). The reasoning is that in RCE, most new rain cells result from thermodynamic pre‐conditioning near the gust front collision lines (Figure 3; see also Fuglestvedt & Haerter, 2020). Besides, the delay between the collision time and the initiation of the resultant rain cell is so large (typically several hours) that direct forced lifting can be ruled out. In line with the findings in Figure 4, CPs with $r<R_{\text{min}}$ are considered too negatively buoyant to initialize new CPs (Drager & van den Heever, 2017; Fournier & Haerter, 2019), and CPs with $r>R_{\text{max}}$ are considered too weak to trigger new events.

The dynamics during the first two generations are introduced in the Methods section (Section 4). After ∼10 generations, new circles are initiated throughout the domain with no obvious patterning (Figure 5a snapshots 4–5). We term this the “high state” having $N_{H}$ circles. Later, a separation into a circle‐filled (convecting) and a circle‐free (nonconvecting) sub‐region occurs (Figure 5a snapshots 6–7). We term this the “low state” having $N_{L}$ circles. Note the visual similarity with the numerical experiment in Figures 1c and 1d. The number of circles $N_{g}$ in all simulations eventually drops from high to low (Figure 5b). The histogram of N, which is bimodal, confirms the notion of two distinct meta‐stable states (Figure 5c). By “meta‐stable state,” we thereby refer to a state resistant to small perturbations but nonresistant to larger perturbations.

We now explore how the two states depend on the independent model parameters $L/R_{\text{min}}$ and $L/R_{\text{max}}$. We find that the number of circles in the low state, $N_{L}$, scales as $N_{L}=L/\left(2R_{\text{min}}\right)$, whereas that in the high state, $N_{H}$, scales as $N_{H}=L^{2}/\left(10R_{\text{min}}^{2}\right)$, both independent of $R_{\text{max}}$ (Figure 5d). The transition point occurs at $N_{T}\approx 1.5L/R_{\text{min}}$. The linear scaling $N_{L}∼L$ is commensurate with band‐like, one‐dimensional structures (compare Figure 5a snapshots 6–7 and Figures 1c and 1d on days 2–4). In contrast, $N_{H}∼L^{2}$ is in line with two‐dimensional organization. By fitting the fraction of simulations in the high state to an exponential function (Figure 5e), we show that a characteristic time exists when the simulations decay to the low state. Thereby, we find that the circle model predicts decreasing $R_{\text{max}}$, increasing L, or increasing $R_{\text{min}}$ speed up the characteristic time when the transition occurs (Figure 5f). Decreasing $R_{\text{max}}$ is in correspondence with the results presented in Figures 1 and 2, increasing L has previously been reported to facilitate self‐aggregation (Bretherton et al., 2005; Muller & Bony, 2015), and rising $R_{\text{min}}$ is due to coarser horizontal resolution (Figures 4e–4h) favoring self‐aggregation (Hirt et al., 2020; Yanase et al., 2020).

## 4 Discussion and Conclusion

There is convincing evidence for the crucial role played by radiative feedbacks in increasing and maintaining a horizontal dry‐moist imbalance in a RCE atmosphere (Bretherton et al., 2005; Emanuel et al., 2014; Muller & Bony, 2015; Muller & Held, 2012; Tompkins, 2001; Wing et al., 2017). In particular, Emanuel et al., (2014) presented a simplified theoretical model for water vapor‐radiation‐circulation feedbacks, in which a linear instability exists that can reinforce an initial moisture imbalance, once formed. Muller and Bony (2015) support this view and highlight the role of clouds and cold pools. Theories have also been proposed for Turing‐instability type coarsening of the RCE atmosphere into moist and dry sub‐regions driven by feedbacks in radiation and surface fluxes (Craig & Mack, 2013). Yet, these classical studies on CSA either use relatively coarse horizontal grid spacing, such as 3 km in Bretherton et al., (2005), or assume the boundary layer moisture to be horizontally homogeneous (Emanuel et al., 2014). These model features lead to weakened or absent representation of CP effects, which are crucial in impacting CSA (Jeevanjee & Romps, 2013). The notion that CP collisions trigger new convective events is well documented (Purdom, 1976; Torri & Kuang, 2019; Weaver & Nelson, 1982) and addressed in toy models (Böing, 2016; Haerter, 2019).

To capture the potential role of CPs during the onset of CSA, we here explicitly model the two‐particle interaction resulting from interacting CP gust fronts. To incorporate the buoyancy suppression effect within the center of each CP, we introduce the radius $R_{\text{min}}$, within which no activation is possible. Such a suppression radius would act against any local positive moisture feedbacks that would favor new rain cells to form close to previous ones. Our study investigates how $R_{\text{min}}$ and the maximal CP radius $R_{\text{max}}$ could influence the ability of an initially scattered rain cell and CP population to eventually facilitate dry regions, which could then grow to give rise to CSA. The circle model implies that large CPs, as formed by pronounced rain evaporation, become space‐filling where CPs fill the whole domain, and there is a connected, percolating patch through the domain among CPs from the same generation. From hexagonal close‐packing, that is, circles organized on a triangular lattice, where each circle exactly touches its six neighboring circles, $L/\left(2R_{\text{max}}\right)$ circles can be placed along one dimension, and $L/\left(\sqrt{3}R_{\text{max}}\right)$ circles can be placed along the other dimension. This gives a lower radius bound for space‐filling $R_{\text{max}}>L{\left(2\sqrt{3}N\right)}^{-1/2}\approx 7.3$ km, where L=96 km is the domain length, and N≈50 is the number of CPs per generation (Figure S5a). For radii smaller than this, areas emerge that newly initialized circles cannot reach, a gap results, and the transition to CSA starts. When (realistically) departing from perfect close‐packing, the required value of $R_{\text{max}}$ is larger, commensurate with our findings (Figure 2) and the transition to CSA between Evap = 0.6, where $R_{\text{max}}$ ≈ 11 km, and Evap = 0.2, where $R_{\text{max}}$ ≈ 8 km.

Similar percolation‐based arguments could be made for cloud‐resolving numerical experiments carried out at coarser horizontal resolution, where CSA was found to be favored compared to fine resolution. At coarse resolution, the number of rain cells may be reduced at the benefit of the rain volume achieved by each rain cell. Percolation may thus be harder to achieve, and dry patches would be more likely to result (Figure S5b). However, we point out that a follow‐up on this point requires careful consideration of how to set a meaningful rain intensity threshold when defining and comparing rain events with differing model resolutions.

Our model simplifies CP expansion by assuming constant radial expansion speed, $v_{0}$. In reality, CPs initially grow quickly, and their expansion speed decreases gradually over a few hours (Figure 2b) (Grant & van den Heever, 2016, 2018). Introducing a smoothly varying gust front speed into our model would require a time‐dependent expansion speed factor, and a numerically approximate approach is more practicable (Haerter et al., 2019). The presented model does not reach a final, fully aggregated state, where a small fraction of the domain intensely convects indefinitely. This sustained activity might be obtained by adding spatial noise (displacing new circles slightly away from the exact geometric collision point) and systematically increased triggering probabilities for decreased overall rain area (Haerter, 2019). Extensions could include explicit incorporation of the “super‐CP” (Windmiller & Hohenegger, 2019) and radiatively driven CP (Coppin & Bony, 2015; Yanase et al., 2020), constituting the two components of the final large‐scale circulation. This model extension would allow triggering events at the edges of the intensely convecting sub‐region due to convective CPs colliding with the opposing radiatively driven CP, a mostly dynamics effect. Such circulation feedbacks may well be essential in stabilizing the final steady‐state, but may not be required to develop the first dry patches and their initial growth, which we have focused on in this work.

Nearly conserved rain cell numbers (Figure S5a) and rain intensities (Figure S2b) are supported by radiation constraints on precipitation in RCE (Held & Soden, 2006). This conservation can be reached by accounting for an additional feedback mechanism: triggering new rain cells by existing CPs may be more dynamic, as the convective instability within the moist convective sub‐region will likely be increased at the expense of the subsiding dry sub‐region. The time delay between CP collisions and dynamical triggering of new convective cells takes O(1h) (Haerter et al., 2019), which is an order of magnitude less than the entire generation time of CPs during the early non‐aggregated state, O(10h) (Figure 4 and Fuglestvedt & Haerter, 2020). Our model could be further developed to explicitly incorporate a time delay between any CP collision and the initiation of the subsequent CP expansion at the location of the collision. For example, this time delay could be chosen proportional to the number of CPs present at a given time.

In conclusion, our simple model captures various characteristics of the onset of convective self‐aggregation (CSA): reduced rain evaporation and larger domain sizes speed up the start of CSA in cloud‐resolving simulations, consistent with reduced $R_{\text{max}}$ and increased L in our circle model. Finally, our model makes the testable prediction that increased suppression radius $R_{\text{min}}$ promotes an early onset of CSA. A corresponding exploration of the parameter space in large‐eddy and cloud‐resolving simulations would be computationally costly, as not only a range of system sizes and rain re‐evaporation rates would need to be explored. Additionally, each parameter combination would require an initial condition ensemble due to the potentially stochastic transition between the high and low CP density states. Our results could guide an exploration of the parameters mentioned. In particular, the exponentially decaying residence likelihood in the high state (Figure 5e) implies a stochastic process, where a transition is possible at equal probability r within each generation, that is, ${\dot{N}}_{H}∼-rN_{H}$. Such a stochastic process could be probed within a reduced set of large‐eddy or cloud‐resolving simulations. If verified, it should be explored, if such a stochastic process also carries over to spatially independent CP processes, at a scale properly chosen to be significantly larger than the typical CP diameter, that is, if cavities can emerge at any sub‐region of the model domain statistically independently of other sub‐regions. Physically, one could alternatively perform a scale analysis of the larger‐scale circulation at different vertical levels to characterize the long‐wavelength modes that are present immediately before the onset of CSA. If such long‐wavelength modes are well below system size, one may be able to conclude that local processes, such as CP effects, indeed constitute the cause of initial dry patch formation.

## Conflict of Interest

The authors declare no conflicts of interest relevant to this study.

## Supporting information

Supporting Information S1

## Data Availability

The large‐eddy simulation data and the source code for the circle model (implemented in MATLAB) are available at http://doi.org/10.5281/zenodo.5228449.
