# Supplementary material for: Circling in on Convective Self‐Aggregation
Source: J Geophys Res Atmos. 2021 Oct 11;126(20):e2021JD035331. doi: 10.1029/2021JD035331 (PMC9285845; doi:10.1029/2021JD035331)
Supplement: Supplementary file 1 — Supporting Information S1 [file JGRD-126-0-s001.pdf]

Supplementary Information for "Circling in on Convective Self-Aggregation"

Silas Boye Nissen[1] and Jan O. Haerter[1,2,3]

[1] *Niels Bohr Institute, University of Copenhagen, Blegdamsvej 17, 2100 Copenhagen, Denmark.*

[2] *Physics and Earth Sciences, Jacobs University Bremen, Campus Ring 1, 28759 Bremen, Germany.*

[3] *Complexity and Climate, Leibniz Center for Tropical Marine Research, Fahrenheitstrasse 6, 28359 Bremen, Germany.*

Contents

1. Text S1.

2. Figures S1–S7.

Introduction

In the supplementary material, we analytically find the average number of circle collisions for neighbors in a system with randomly positioned cells (Text S1). Besides, we provide supplementary figures, including the initial conditions for the large-eddy simulations (LES) (Fig. S1), the time development of the domain-mean specific humidity variation, rain intensity, and temperature (Fig. S2), the independence on the initial number of circles (Fig. S3), the numerical replication rate in the circle model (Fig. S4), the number of rainfall tracks in the LES data (Fig. S5), the nearest neighbor distance data contrasted to the control (Fig. S6), and an illustration of the line-of-sight definition (Fig. S7).

Text S1. The number of circles in generation 2 when $R_{\mathbf{min}} = 0$ and $R_{\mathbf{max}} = \infty$.

We seek to compute the number of *direct* circle collisions for an initial random population of circle centers, with circles emerging synchronously and at equal speed from all circle centers. By *direct*, we mean that the collision takes place along the line connecting the two circle centers involved. Finding the number of such collisions is equivalent to finding the number of *line-of-sight* connections (also known as Gabriel connections) among these points (Fig. S7).

Consider N points randomly seeded in a total area $A = \pi R^2$. A line-of-sight connection between any two points $\mathbf{r_i}$ and $\mathbf{r_j}$ at distance $l \equiv |\mathbf{r_i} - \mathbf{r_j}|$ exists if no points are located inside the circle of radius $l/2$ centered at $(\mathbf{r_i} + \mathbf{r_j})/2$ (Fig. S7). This is the type of connection that we require for any two colliding circles to initiate a new expanding circle in Fig. 5. One must further consider the probability of finding two points at distance l. For this purpose, define the density of points as $\rho \equiv N/A = N/\pi R^2$. Now consider the infinitesimal area $a(l) \equiv 2\pi\, l\, dl$ between two circles of radii l and $l + dl$. The number of points contained in this area is

$$n(l)\, dl = \rho\, a(l) = \frac{2Nl}{R^2}\, dl. \tag{S1}$$

For any two points at a given distance l, we now consider the probability $p(l)$, that none of the remaining $N - 2$ points lie within the circle of radius $l/2$:

$$P(l) = P_0^{N-2} = \left(1 - \frac{\pi(l/2)^2}{\pi R^2}\right)^{N-2}, \tag{S2}$$

where

$$P_0 = 1 - \frac{\pi(l/2)^2}{\pi R^2} \tag{S3}$$

is the probability that any single point is not inside the area enclosed by a circle of radius $l/2$. Now the total number of expected line-of-sight (LOS) connections for a fixed given point to any of the other points can be computed:

$$N_{\mathrm{LOS}} = \int_0^R dl\, n(l) P(l) \tag{S4}$$

$$= \int_0^R dl\, \frac{2Nl}{R^2} \left(1 - \frac{l^2}{4R^2}\right)^{N-2} \tag{S5}$$

$$= \frac{4(1 - \left(\frac{3}{4}\right)^{N-1})}{1 - N^{-1}}, \tag{S6}$$

which gives $\lim_{N\to\infty} N_{\mathrm{LOS}} = 4$. Hence, when repeating for all N and avoiding double-counting connections, one obtains $2N$ line-of-sight connections when proceeding from generation 1 to 2 for $R_{\min} = 0$ and $R_{\max} = \infty$. In Fig. S4, we numerically confirm this replication rate, $r_1 = 2$, and evaluate later generations numerically.

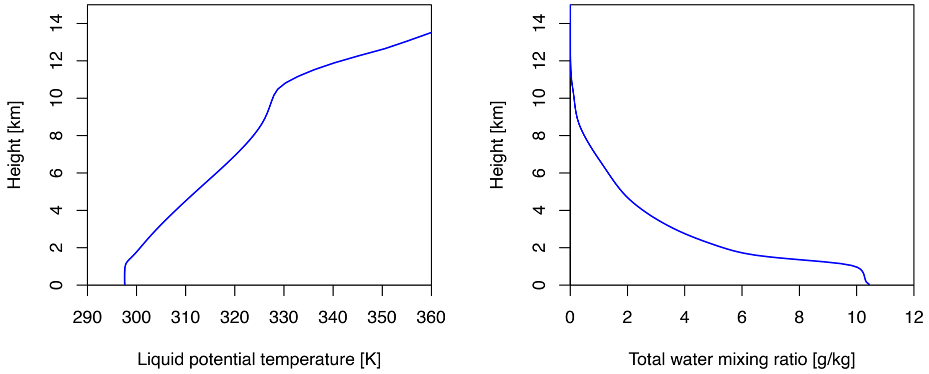

FIG. S1. **Initial condition.** Vertical profiles for temperature (left) and moisture (right) used as the initial condition for all numerical experiments.

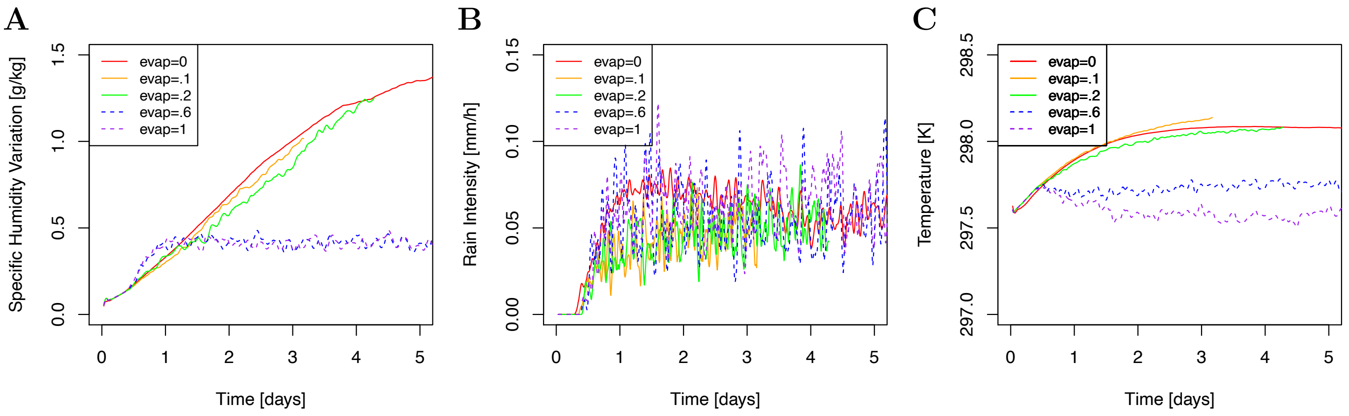

FIG. S2. **Low-level humidity variation, domain mean rainfall, and temperature**. **(A)** Each curve (*see* legend for line style) was computed from the low-level ($z = 50$ m) difference $\Delta q(t) \equiv q_{v,75}(t) - q_{v,25}(t)$, where the subscript numbers denote the respective percentiles of the specific humidity for each simulation and the argument t denotes the given simulation output time step. The continued increase for Evap $\in \{0, 0.1, 0.2\}$ signals the onset of self-aggregation for those cases. **(B)** Domain-mean low-level ($z = 50$ m) rain intensity for each simulation over time. **(C)** Domain-mean low-level ($z = 50$ m) temperature for each simulation over time. We attribute the kink for the dis-aggregated simulations (Evap=0.6 and Evap=1) near $t = 0.5$ days to the onset of rainfall. Throughout the figure, the horizontal resolution in the RCE simulations is 200 m.

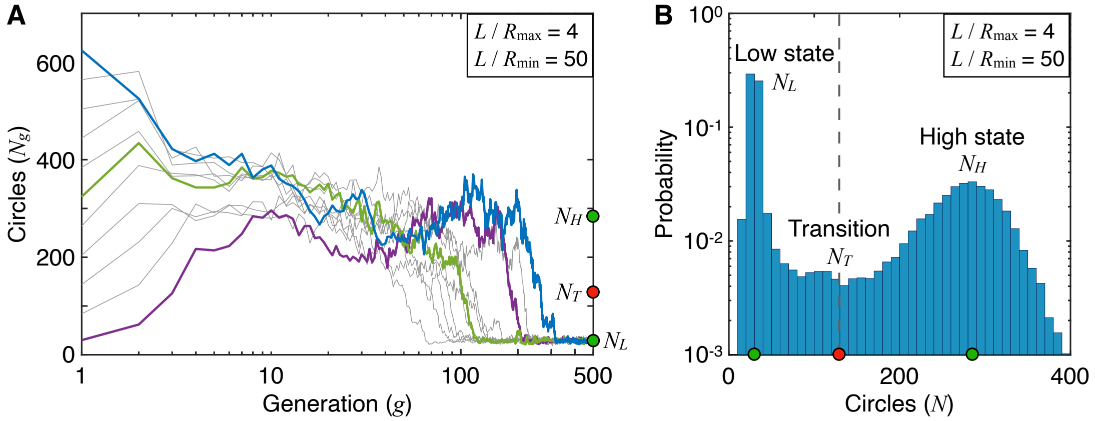

FIG. S3. **The circle model (presented in Fig. 5) is not sensitive to the initial circle number (called N_1). (A)** Here, we show 11 model runs with varying N_1. Note, a logarithmic x-axis is applied to show how all runs reach the high state, N_H, within ten generations independent of N_1. Otherwise, similar to Fig. 5B. **(B)** Similar to Fig. 5C with the difference that we now pool together 100 runs all varying N_1. Note that the low state, N_L, the transition point, N_T, and the high state, N_H, all locate at the same number of circles as obtained in Fig. 5C.

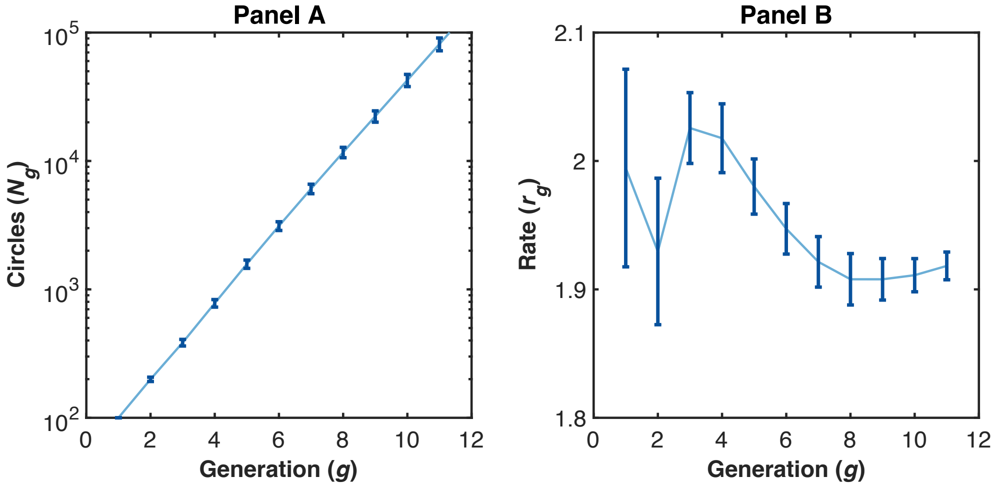

FIG. S4. **Circle model when $R_{\mathbf{min}} = 0$ and $R_{\mathbf{max}} = \infty$. (A)** The number of circles (N_g) as a function of generation (g). Error bars indicate standard deviation obtained from 20 runs, all starting with $N_1 = 100$ circles. Note the logarithmic y-axis. **(B)** The replication rate ($r_g = N_{g+1}/N_g$) of the runs in (A). Note that $r_1 = 2$ as found analytically in the supplementary text.

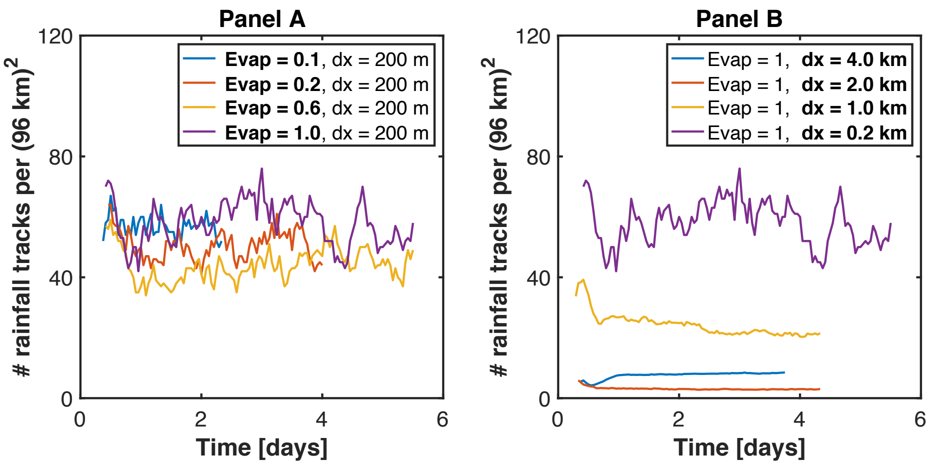

FIG. S5. **Time series of the number of rainfall tracks in all RCE simulations performed. (A)** The number of tracks within a 6-hour running time window when keeping the horizontal resolution $dx = 200$ m constant and varying the evaporation rate (corresponding to the simulations presented in Fig. 4A–D). **(B)** The same when changing the horizontal resolution and keeping the evaporation rate Evap=1 constant (corresponding to the simulations presented in Fig. 4E–H). Note that the purple line represents the same simulation in the two panels. In (B), we normalize the rainfall track number to the domain size $L = 96$ km used for the 200 m horizontal resolution simulations. We note that these numbers are only easily compared for equal-resolution simulations since detecting a rainfall track is done by setting an intensity threshold for each pixel. For fine resolution cases, there will be many small pixels that exceed the threshold. Whereas the opposite applies to coarse resolution.

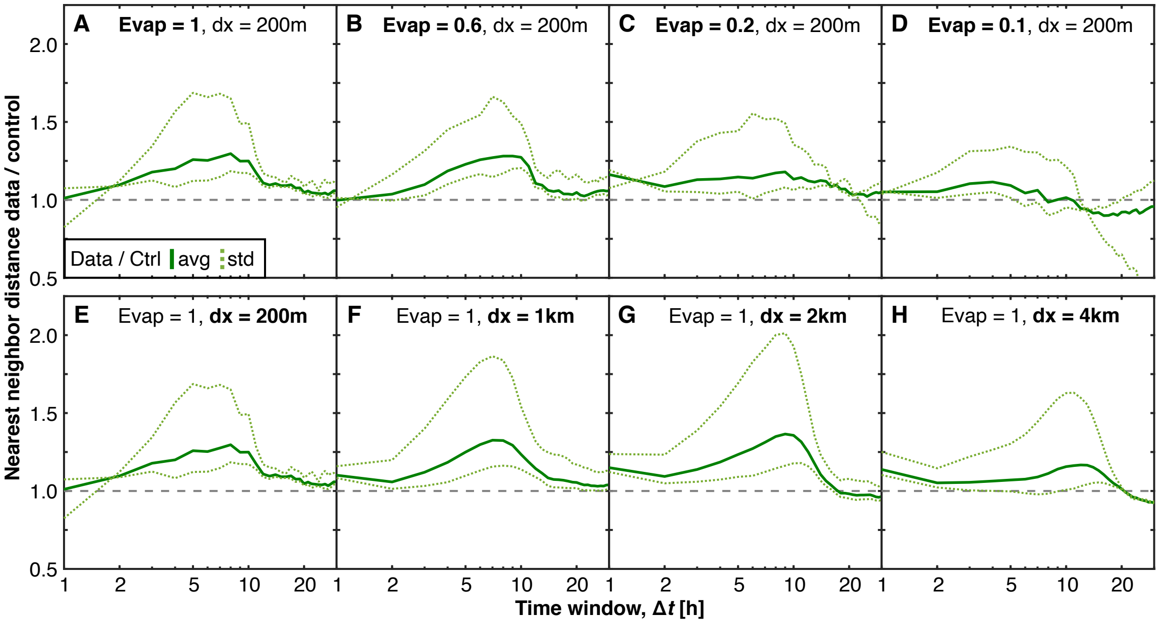

FIG. S6. **Comparing the nearest neighbor distance in RCE data to a uniform distribution of points.** Here we obtain the green lines by dividing the blue lines with the red lines in Fig. 4. Solid green lines show average results, and dashed green lines indicate standard deviations. The horizontal dashed gray line marks 1.0.

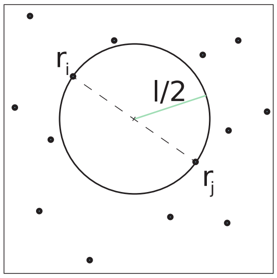

FIG. S7. **A line-of-sight connection.** Schematic illustrating points in a 2D domain. Two points, $\mathbf{r}_i$, and $\mathbf{r}_j$, separated by a distance l, have a line-of-sight connection given that no points are located inside the circle of radius $l/2$ with the two points on its rim. In mathematics, this concept is known as Gabriel neighbors.
